# The Combinational Polymorphisms of *ORAI1* Gene Are Associated with Preventive Models of Breast Cancer in the Taiwanese

**DOI:** 10.1155/2015/281263

**Published:** 2015-08-25

**Authors:** Fu Ou-Yang, Yu-Da Lin, Li-Yeh Chuang, Hsueh-Wei Chang, Cheng-Hong Yang, Ming-Feng Hou

**Affiliations:** ^1^Graduate Institute of Medicine, College of Medicine, Kaohsiung Medical University, Kaohsiung 80708, Taiwan; ^2^Cancer Center, Kaohsiung Medical University Hospital, Kaohsiung Medical University, Kaohsiung 80708, Taiwan; ^3^Institute of Clinical Medicine, Kaohsiung Medical University, Kaohsiung 80708, Taiwan; ^4^Department of Surgery, Kaohsiung Municipal Ta-Tung Hospital, Kaohsiung, Taiwan; ^5^Department of Electronic Engineering, National Kaohsiung University of Applied Sciences, Kaohsiung 80778, Taiwan; ^6^Department of Chemical Engineering & Institute of Biotechnology and Chemical Engineering, I-Shou University, Kaohsiung 84001, Taiwan; ^7^Institute of Medical Science and Technology, National Sun Yat-sen University, Kaohsiung 80424, Taiwan; ^8^Research Center of Environmental Medicine, Kaohsiung Medical University, Kaohsiung 80708, Taiwan; ^9^Department of Biomedical Science and Environmental Biology, Kaohsiung Medical University, Kaohsiung 80708, Taiwan; ^10^National Sun Yat-Sen University-Kaohsiung Medical University Joint Research Center, Kaohsiung 80424, Taiwan; ^11^Department of Biological Science and Technology, National Chiao Tung University, Hsinchu 30010, Taiwan

## Abstract

The ORAI calcium release-activated calcium modulator 1 (*ORAI1*) has been proven to be an important gene for breast cancer progression and metastasis. However, the protective association model between the single nucleotide polymorphisms (SNPs) of *ORAI1* gene was not investigated. Based on a published data set of 345 female breast cancer patients and 290 female controls, we used a particle swarm optimization (PSO) algorithm to identify the possible protective models of breast cancer association in terms of the SNPs of *ORAI1* gene. Results showed that the PSO-generated models of 2-SNP (rs12320939-TT/rs12313273-CC), 3-SNP (rs12320939-TT/rs12313273-CC/rs712853-(TT/TC)), 4-SNP (rs12320939-TT/rs12313273-CC/rs7135617-(GG/GT)/rs712853-(TT/TC)), and 5-SNP (rs12320939-TT/rs12313273-CC/rs7135617-(GG/GT)/rs6486795-CC/rs712853-(TT/TC)) displayed low values of odds ratios (0.409–0.425) for breast cancer association. Taken together, these results suggested that our proposed PSO strategy is powerful to identify the combinational SNPs of rs12320939, rs12313273, rs7135617, rs6486795, and rs712853 of *ORAI1* gene with a strongly protective association in breast cancer.

## 1. Introduction

Single nucleotide polymorphisms (SNPs) are the most common variants of human genome [1]. Genome-wide association studies (GWAS) have widely been used to detect the association models to diseases in terms of multiple SNPs [2–7]. The SNP interaction was gradually identified in a lot of GWAS [8–10] and non-GWAS [11, 12] literature.

The ORAI calcium release-activated calcium modulator 1 (*ORAI1*) [13] was reported to be involved in cancer progression and metastasis of several types of cancers [14–17]. The cell- and animal-based studies found that inhibition of* ORAI1* gene impeded the migration of breast cancer cells [18]. Several association studies of the SNPs of* ORAI1* gene were also investigated in predicting the predisposition of diseases and cancers [19–22]. However, the SNP-SNP interaction-based association model between SNPs of* ORAI1* gene and the protective association in breast cancer was less addressed.

For computational biologic challenge, the significant and potential association models are usually hidden in the large number of possible combinations between several genotypes of SNPs. Many methods had been developed to analyze the potential association models to GWAS using the traditional statistics, data mining, and machine learning techniques [23–30]. Among them, the particle swarm optimization (PSO) method was used to explore the association models for several diseases and cancers [28]. The advantages of PSO are easy and rapid to apply the statistics analysis to identify the potential association models.

The objective of this study aims to use the PSO to investigate whether combinational SNPs of* ORAI1* gene in data set [22] are protectively associated with breast cancer in the Taiwanese population.

## 2. Methods

### 2.1. Problem Description

The set *X*_*i*_ = {*x*_*i*1_, *x*_*i*2_,…, *x*_*iD*/2_, *x*_*iD*/2+1_,…, *x*_*iD*_}, including SNP combinations {*x*_*i*1_, *x*_*i*2_,…, *x*_*iD*/2_} with their corresponding genotypes {*x*_*iD*/2+1_,…, *x*_*iD*_}, is defined as possible solution in the detection of protective association model problem, and the set is named SNP barcode in this study. The objective function (fitness function) *f*(*X*_*i*_) is defined as the difference between case group and control group. The objective of detecting the protective association model is a search for maximal SNP barcode *X* ^*∗*^ via the evaluation of objective function *f*(*X*)  (*f* : *δ*⊆*ℛ* ^*D*^ → *ℛ*); that is, *f*(*X* ^*∗*^) > *f*(*X*) for all *X* ∈ *δ*, where *δ* is a nonempty large finite set serving as the search space, and *δ* = *ℛ* ^*D*^.

### 2.2. PSO

In PSO, particle is regarded as a solution of any problem [31]. The two experiences, (1) the particle's own experience (*pbest*) and (2) the global knowledge (*gbest*), are the two important objectives for leading the particle moves toward better search region of the problem space. An optimal result can be searched by* gbest* when the PSO produce is repeated in much generation.

Algorithm 1 illustrates the PSO produce which has the four operations, including particle initializations, particle evaluations,* pbest* and* gbest* updates, and particle position update. The first step initializes the particles reasonable values. The second step computes the fitness values of particles. The third step updates the* pbest* of particle if the fitness value is better than the* pbest*. The fourth step updates the* gbest* if a fitness value of particle is better than the* gbest*. The fifth step updates the particle's velocity and position. The steps 2 to 5 are repeated until the maximum generation is achieved. Next, these four operations are introduced in detail as follows.

### 2.3. Particle Initializations

A particle is defined as the SNP barcode; that is, *X*_*i*_ = {*x*_*i*1_, *x*_*i*2_,…, *x*_*iD*/2_, *x*_*iD*/2+1_,…, *x*_*iD*_}. The initial population (i.e., generation is 0) should cover this range as much as possible by randomizing individuals within the problem space constrained by the prescribed minimum and maximum bounds: *X*_*i*,min_ = {*x*_*i*,1,min_, *x*_*i*,2,min_,…, *x*_*i*,*D*,min_} and *X*_*i*,max_ = {*x*_*i*,1,max_, *x*_*i*,2,max_,…, *x*_*i*,*D*,max_}. The *j*th element of the *i*th particle can initialize as

(1) xi,j,0=xi,j,min+randi,j0,1·xi,j,max−xi,j,min,xj,max=SNPmax,j≤D2∑xGenotypemax,j>D2,xj,min=SNPmin,j≤D2∑XXGenotypemin,j>D2,Genotype=1,recessive  genotype2,dominant/heterozygous  genotype,

where SNP_max_ and SNP_min_ are the maximum number of SNPs and the minimum number of SNPs, respectively. Genotype_min_ is set to 1 (i.e., the minor allele is regarded as the recessive genotype) and Genotype_max_ is set to 2 (i.e., the major allele is regarded as the dominant genotype with the homologous major genotype or heterozygous genotype).

### 2.4. Particle Evaluations

The fitness function is defined by the frequency difference value between breast cancer patients and controls, and the relevant equation can be written as

(2) $$\begin{array}{c} f\left(X_{i}\right)=\frac{\left(X_{i}\cap control\right)}{controls}-\frac{\left(X_{i}\cap breast\;patients\right)}{patients}. \end{array}$$

The *X*_*i*_ represents the *i*th particle. The *X*_*i*_∩control is defined as the total number of intersections between the *i*th particle and control group. The controls are defined as the total number of control group. The *X*_*i*_∩breast  patients is defined as the total number of intersections between the *i*th particle and breast patient group. The patients are defined as the total number of breast patient group.

### 2.5. *pbest* and* gbest* Updates

The* pbest* can record the particle experience, and* gbest* can record the common experience of particles. For* pbest* update, if the current fitness value of particle is better than* pbest*, then both the position and fitness values of* pbest* are replaced by the current position and fitness values of this particle. For* gbest* update, if the fitness value of* pbest* is better than that of* gbest*, then both the position and fitness values of* gbest* are replaced by the current position and fitness values of* pbest*.

### 2.6. Particle Position Update

The particle position is updated by the three different vectors, including the inertia weight *w*,* pbest*, and* gbest*. Equation (3) is the *w* updating function, and this function can iteratively reduce the value of *w* from *w*_max_ to *w*_min_ [33]. Equation (4) is used to update the particle velocity. Equation (5) is used to adjust the particle position. Consider

(3) $$\begin{array}{c} w_{LDW}=\left(w_{max}-w_{min}\right)\times \frac{Iteration_{max}-Iteration_{i}}{Iteration_{max}}+w_{min}, \end{array}$$

(4) vidnew=wLDW×vidold+c×r1×pbestid−xidold +c×r2×gbestd−xidold,

(5) $$\begin{array}{c} x_{id}^{new}=x_{id}^{old}+v_{id}^{new}, \end{array}$$

where *w*_max_ is maximum value of inertia weight *w* and *w*_min_ is minimum value of inertia weight *w*. Iteration_max_ is the maximum generation. The *r*_1_ and *r*_2_ are the random functions within the range [0, 1]. The acceleration constants *c*_1_ and *c*_2_ are used to control the particle search direction (*pbest* or* gbest*). Velocities *v*_*id*_^new^ and *v*_*id*_^old^ are the new and old velocities, respectively. The *x*_*id*_^old^ and *x*_*id*_^new^ are the current and updated particle positions, respectively.

### 2.7. Parameter Settings

In this study, the PSO parameters are chosen under the optimal setting [34]. For example, the population size is 50, the maximum generation is 100, the *w*_max_ of the inertia weight *w* is 0.9, the *w*_min_ is 0.4 [33], *V*_max_ is set to (*X*_max_ − *X*_min_), and *V*_min_ is set to −(*X*_max_ − *X*_min_). Learning factors *c*_1_ and *c*_2_ are both set to 2 [35].

### 2.8. Data Set Collection

In this study, we selected the five* ORAI1* related SNPs from the HapMap Han Chinese database, including rs12320939, rs12313273, rs7135617, rs6486795, and rs712853, and the breast cancer data set with patients (*n* = 345) and controls (*n* = 290) were obtained from our previous study [22].

### 2.9. Statistical Analysis

The odds ratio (OR), 95% confidence interval (CI), and *P* value were used to evaluate the detected association models. A *P* value < 0.05 indicates the occurrence of the association models significantly differing between the breast cancer patients and controls. The SPSS version 19.0 (SPSS Inc., Chicago, IL) was used to compute all statistical analysis.

## 3. Results

### 3.1. Evaluation of the Breast Cancer Risk of Individual SNP

Table 1 showed the breast cancer risks of five individual SNPs in* ORAI1* gene. Among them, we identified six genotypes of SNPs with the protective association against breast cancer, including rs12320939-TT, rs12313273-CC, rs7135617-TT, rs6486795-CC, and rs712853-CC. However, the frequency differences of these genotypes for each individual SNP were nonsignificant between the breast cancer patients and controls.

### 3.2. The Association Models of 2-SNP Combinations with Maximum Differences between Cases and Controls

Table 2 showed the top ten association models of 2-SNP combinations from five SNPs listed in Table 1. Four association models showed significant difference between paired specific combination and others (*P* < 0.05), including SNPs (1-2)-genotypes (1-1), SNPs (2-4)-genotypes (1-1), SNPs (2-3)-genotypes (1-2), and SNPs (2-5)-genotypes (1-2). In these 2-SNP association models, the SNPs (1-2)-genotypes (1-1), that is, [rs12320939-TT]-[rs12313273-CC], had the maximum frequency difference (5.65%) between the breast cancer patients and controls and displayed the smallest OR value (<1) with a protective effect against breast cancer. Similarly, the SNPs (1-2)-genotypes (1-1) displayed the highest power value between these models of 2-SNP combinations.

### 3.3. The Association Models of 3- to 5-SNP Combinations with Maximum Differences between Cases and Controls

Using similar computation like in Table 2, Table 3 showed the best association models of 3- to 5-SNP combinations with maximum difference between the breast cancer patients and controls. We found that three SNPs rs12320939, rs12313273, and rs712853 were strongly associated with protective effect against breast cancer when their genotypes were TT, CC, and TT/TC, respectively (OR = 0.409, 95% CI = 0.215–0.779, *P* = 0.005). The 4-SNP combinations showed that rs7135617 was included to generate the protective association with breast cancer. The OR, *P* value, and power were the same for 3-, 4-, and 5-SNP combination models. For 5-SNP model, SNPs (1, 2, 3, 4, 5) showed a similar protective effect against breast cancer when their genotypes are TT, CC, GG/GT, CC, and TT/TC, respectively (OR = 0.425, 95% CI = 0.223–0.813, *P* = 0.008).

## 4. Discussion

SNP interaction analyses can improve the performance of association studies in disease predisposition [26, 36–41]. In this study, we investigated the protective factors for genetic variants of complex traits in breast cancer. We hypothesized that five important SNPs within the* ORAI1* gene may reduce the genetic susceptibility to breast cancer. In the current study, a robust PSO algorithm combined with the statistical analysis was used to detect the relationship between protective association of breast cancer and* ORAI1* SNPs. As expected, our proposed PSO algorithm has a good performance to identify the protective effects of* ORAI1* SNPs against breast cancer in this study.

The statistical analyses were reported to have the difficulty to identify the complex multifactor association [42]. Accordingly, several studies proposed comprehensive approaches to identify the association model with disease related factors [27, 30, 43, 44]; these approaches have adequate power to explore the potential association models. The SNP combination generated by PSO can detect the association relationship in terms of selecting several important genotypes of SNPs. This algorithm can help us to understand the genetic basis of the complex diseases/traits.

Our previous studies had shown that* ORAI1* is an associated gene to breast cancer with the nodal involvement, progesterone receptor status, and estrogen receptor status studies [22]. In our previous work [32], the specific combinational SNPs of* ORAI1* gene were reported to be associated with breast cancer risk. However, the protective association of breast cancer in terms of combinational SNPs of* ORAI1* gene was not investigated in SNP-SNP interaction manner. In the current study, we found a strong protective association between specific combinational SNPs of* ORAI1* gene in relation to breast cancer susceptibility.

We detected the possible 2-factor association models in terms of specific SNP combination. PSO analysis selected two SNPs (rs12320939 and rs12313273) in* ORAI1* genes as the best protective association model against breast cancer when the genotypes of rs12320939 and rs12313273 are TT and CC, respectively. This model can not specify whether the model was a synergistic relationship or not, but it suggested that the combination of factors (rs12320939 with genotype TT and rs12313273 with genotype CC) had very low risk for breast cancer susceptibility.

Haplotype is defined by a group of heritable SNPs of linked genes on the same chromosome. Haplotype analysis can provide the performance between cases and controls for patterns of SNP combination involving all SNPs, for example, 5 SNPs in the case of the current study. However, the SNP-SNP interactions for different SNPs involved are not considered in traditional haplotype analysis. In contrast, our proposed PSO-based SNP-SNP interaction was not limited to SNPs of the same chromosome although it is in the current study. Moreover, our proposal algorithm can identify the best SNP model with the maximum difference between cases and controls for different numbers of SNPs, for example, from 2 to 5 SNPs. Recently, haplotype analysis was also reported to combine with PSO [45, 46]. Therefore, the computation of traditional haplotype analysis may be improved with the help of PSO.

## 5. Conclusions

We used the PSO strategy to detect the protective association models between five combinational SNPs of* ORAI1* gene in the breast cancer. Among them, the two SNPs (rs12320939 and rs12313273) were found to be most essential components to protectively associate in breast cancer when their genotypes are TT and CC, respectively. PSO identified SNP model may enhance the detection of genetic variants to disease or cancer susceptibility. Therefore, our findings provided the important information regarding combinational patterns of SNPs located in the relevant genes.

## Figures and Tables

**Algorithm 1:**
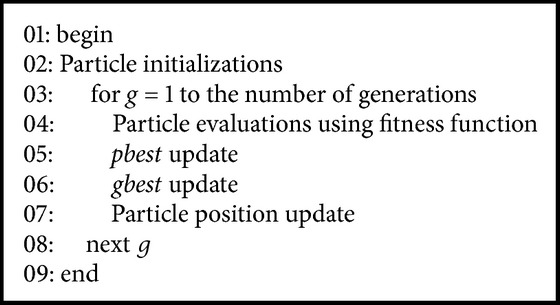
Particle swarm optimization pseudocode.

**Table 1:** Estimated risk of each individual SNP on the occurrence of breast cancer.

SNPs	Genotype	Breast cancer patients (%)^∗1^	Controls (%)^∗1^	OR (95% CI)^∗2^
		(*n* = 345)	(*n* = 290)	
rs12320939	(1) TT	67	71	0.74 (0.51–1.09)
	(2) GG/GT	278	219	1

rs12313273	(1) CC	20	29	0.55 (0.31–1.00)
	(2) TT/TC	325	261	1

rs7135617	(1) TT	55	51	0.89 (0.59–1.35)
	(2) GG/GT	290	239	1

rs6486795	(1) CC	35	43	0.65 (0.40–1.04)
	(2) TT/TC	310	247	1

rs712853	(1) CC	33	28	0.99 (0.58–1.68)
	(2) TT/TC	312	262	1

^*∗*1^The genotype information of case and control was derived from our previous work [32] and it was reachable at http://bioinfo.kmu.edu.tw/BRCA-ORAI1-5SNPs.xlsx.

^∗2^The genotype frequencies on the occurrence of breast cancer are not significant (*P* > 0.05).

OR = odds ratio.

**Table 2:** The top ten best protective association models of 2-SNP combinations.

Specific 2-SNP combination^∗1^	Genotypes^∗1^	Case number /control number	OR	95% CI	*P* value	Power
1-2	Others	330/261	1		0.005^∗2^	0.792
	1-1	15/29	0.409	0.215–0.779		

2-4	Others	330/261	1		0.010^∗2^	0.752
	1-1	15/28	0.424	0.222–0.810		

1-3	Others	278/219	1		0.123	0.339
	1-2	67/71	0.743	0.509–1.085		

2-3	Others	327/261	1		0.022^∗2^	0.629
	1-2	18/29	0.495	0.269–0.912		

1-4	Others	310/247	1		0.073	0.432
	1-1	35/43	0.649	0.403–1.044		

3-4	Others	310/247	1		0.073	0.432
	2-1	35/43	0.649	0.403–1.044		

4-5	Others	311/249	1		0.096	0.385
	1-2	34/41	0.664	0.409–1.078		

2-5	Others	325/261	1		0.048^∗2^	0.506
	1-2	20/29	0.554	0.306–1.002		

1-5	Others	289/234	1		0.311	0.174
	1-2	56/56	0.810	0.538–1.218		

2-3	Others	292/239	1		0.451	0.118
	2-1	53/51	0.851	0.558–1.296		

^*∗*1^The information of the SNP and genotypes is provided in Table 1.

^∗2^The models have significance on the occurrence of breast cancer (*P* < 0.05).

OR = odds ratio.

**Table 3:** Estimated joint effects on models of 2- to 5-SNP combinations associated with breast cancer.

Combined SNP number (specific SNP combination)^∗1^	SNP genotypes	Case number /control number	OR	95% CI	*P* value	Power
2-SNP	Others	330/261	1		0.005^∗2^	0.792
(1-2)	1-1	15/29	0.409	0.215–0.779		
3-SNP	Others	330/261	1		0.005^∗2^	0.792
(1-2-5)	1-1-2	15/29	0.409	0.215–0.779		
4-SNP	Others	330/261	1		0.005^∗2^	0.792
(1-2-3-5)	1-1-2-2	15/29	0.409	0.215–0.779		
5-SNP	Others	330/262	1		0.008^∗2^	0.750
(1-2-3-4-5)	1-1-2-1-2	15/28	0.425	0.223–0.813		

^*∗*1^The information of the SNP and genotypes is provided in Table 1.

^∗2^The models have significance on the occurrence of breast cancer (*P* < 0.05).

OR = odds ratio.
